# Deep learning-assisted terahertz intelligent detection and identification of cancer tissue

**DOI:** 10.1016/j.fmre.2025.03.013

**Published:** 2025-03-26

**Authors:** Xingyu Wang, Yafei Xu, Rong Wang, Nuoman Tian, Zhengpeng Zhu, Shuting Fan, Liuyang Zhang, Ruqiang Yan, Xuefeng Chen

**Affiliations:** a State Key Laboratory for Manufacturing Systems Engineering, Xi'an Jiaotong University, Xi'an 710049, China; b Zhengzhou Research Institute, Harbin Institute of Technology, Zhengzhou 450000, China; c Department of Pathology, Sinopharm Dongfeng General Hospital, Hubei University of Medicine, Shiyan 442008, China; d Key Laboratory of Optoelectronic Devices and Systems of Ministry of Education and Guangdong Province, College of Physics and Optoelectronics Engineering, Shenzhen University, Shenzhen 518060, China

**Keywords:** Terahertz detection technique, Cancer diagnosis, Artificial intelligence, Automatic and intelligent diagnosis, Terahertz time-domain spectroscopy

## Abstract

Cancer, as one of the most notorious health diseases, represents the main reason attributed to millions of worldwide deaths each year. Timely detection and accurate diagnosis are thus vital to cancer prevention and timely therapy. Traditional cancer prescreening is not only cumbersome but also heavily reliant on sufficient expert knowledge, which inevitably increases the complexity of cancer diagnosis and limits early cancer diagnosis. To overcome this problem, recent terahertz (THz) technology, as an unconventional bio-friendly detection approach, has emerged with great potential in human disease diagnosis due to its non-ionic and high-resolution features. By combining the THz detection technique and artificial intelligence technique, here we propose a dense and efficient channel attention network (DECANet) framework-based THz diagnosis system for cancer prescreening. The cancer identification and diagnosis process are transformed into one end-to-end classification process of THz signals reflected from cancer tissue. The biosamples of breast and skin cancer tissue are characterized to validate the effectiveness and applicability of the proposed approach. Our quantified results indicate that our proposed THz diagnosis framework has promising feature extraction capability for abnormal cancerous tissue and provides an effective complement tool to assist the healthcare cancer diagnosis.

## Graphical abstract

**Figure:**
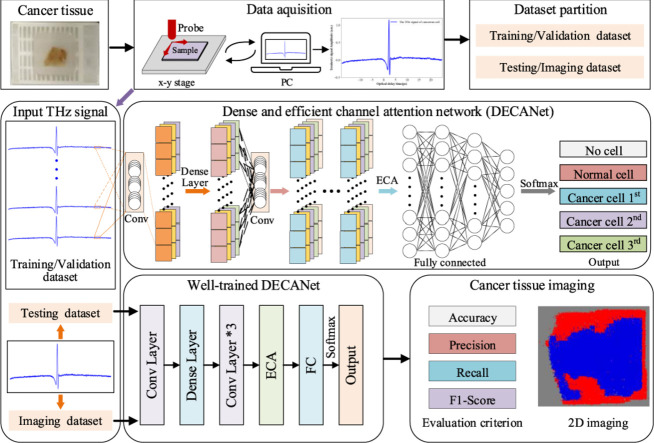

## 1 Introduction

Cancer has risen to become the second most general cause of mortality among humans following cardiovascular diseases in the world [1]. In current clinical examination, conventional cancer initial screening methods, such as genetic testing [2], imaging [3] and cancer pathology [4], etc., play a dominant role in cancer diagnosis and treatment. Among them, genetic testing requires sophisticate testing techniques and data analysis. Imaging methods such as extradosed CT [5] and X-ray [6] may be subjected to ionizing radiation risk to the human body. Histological examination needs to complete a series of lengthy pathological examinations such as sample collection, fixation, paraffin embedding, sectioning, staining and microscopic observation. Therefore, the accurate screening of human cancer tissue is of great significance for early cancer diagnosis and prevention [7].

Recently, terahertz (THz) technology has shown great potential as an emerging bio-friendly detection approach in the field of human disease diagnosis [8]. THz wave is located between infrared light and microwaves in the electromagnetic spectrum, spanning frequencies from 0.1 to 10 terahertz. Compared to X-ray detection techniques, non-ionizing THz radiation has lower penetration capabilities but interacts more safely with biological tissues and molecules, providing unique molecular information [9]. By harnessing THz power, it comes to the reality to examine and diagnose diseases without imposing any damage to biological samples [10,11]. The identification process of diseased tissue based on THz technology can be isolated from the objective evaluation of physical doctors and provide a subjective reference for cancer diagnosis [12]. In recent decades, researchers have intended to employ terahertz time-domain spectroscopy (THz-TDS) in the field of cancer detection, leveraging the high water content in cancerous tissues as a potential marker for malignant tumors. In 2003, THz pulse imaging was firstly employed to analyze basal cell carcinoma in ex vivo studies [13], revealing higher absorption in diseased tissue due to increased interstitial water or changes in water molecule vibrations. In 2004, Woodward et al. [14] demonstrated the potential of THz pulse imaging to delineate tumor margins, and in 2007, Hoshina et al. [15] applied chemometrics to THz-TDS for analyzing multiple tumor samples. In 2016, Rahman et al. [16] utilized THz-TDS to unveil significant and quantifiable differences in ex-vivo between the healthy and basal cell carcinoma skin samples. In 2022, Ke et al. [17] explored the application of THz-TDS for diagnosing pathological resection margins in laryngeal cancer.

Although THz-TDS has been gradually utilized in cancer diagnosis due to its unique advantages, the susceptibility of terahertz signals to various complex interferences such as noise, dispersion, and overlap often leads to decreased detection accuracy. Additionally, the presence of a substantial number of water molecules in biological tissues limits THz waves due to water absorption. So numerous signal processing methods have previously been applied to derive desirable features. For example, Liu et al. [18] have used THz detection and wavelet entropy feature extraction method to detect breast invasive ductal carcinoma. Wu et al. [19] have proposed a multinomial Bayesian algorithm for breast cancer detection via THz imaging of fresh murine tumors.

However, these signal-processing techniques require professional knowledge and experience and might be difficult to meet the demanding needs of automatic and intelligent cancer diagnosis. Additionally, ML has been extensively employed in THz medical image processing, including automatic segmentation of cancer areas in pathology images of cancer tissue. Chavez et al. [20] applied unsupervised Bayesian learning algorithm to THz images for classifying the different tissue regions in xenograft breast tumors. However, such studies necessitate the utilization of stained pathological tissue images. Subsequently, the color and morphological features in the pathological images are harnessed for the segmentation of cancerous tissues in THz images.

As a significant field within ML, deep learning (DL) employs multi-layer neural networks to automatically extract the features from chaos signals [21,22]. In addition, deep learning [23] can efficiently process large datasets by distributed computing and Graphics Processing Unit (GPU) technology. Currently, some researchers have implemented deep learning algorithms on THz images to realize the cancer diagnosis. For example, Liu et al. [21] have utilized deep learning models to classify breast tissue captured through THz imaging. However, it remains large room to improve the detection accuracy.

Therefore, we have combined THz detection technology with artificial intelligence techniques in the field of cancer tissue detection to fulfill the accurate identification of cancer tissue. We propose a THz cancer diagnostic system named DECANet framework to distinguish THz signals from normal tissues and cancer tissues and simplify cancer identification into a classification problem of collected THz signals from cancer tissues. Our research can skip subsequent steps in pathological examination, such as staining and microscopic observations, to further improve the cancer detection efficiency, and show that water content is not the exclusive origin of contrast within the terahertz wave spectrum. Additionally, a series of experiments have demonstrated that our proposed THz diagnostic system has strong feature extraction capabilities and can successfully perform automatic cancer diagnosis on cancerous human tissues.

## 2 Materials and methods/experiment

### 2.1 Reflected THz-TDS system

**Fig. 1:**
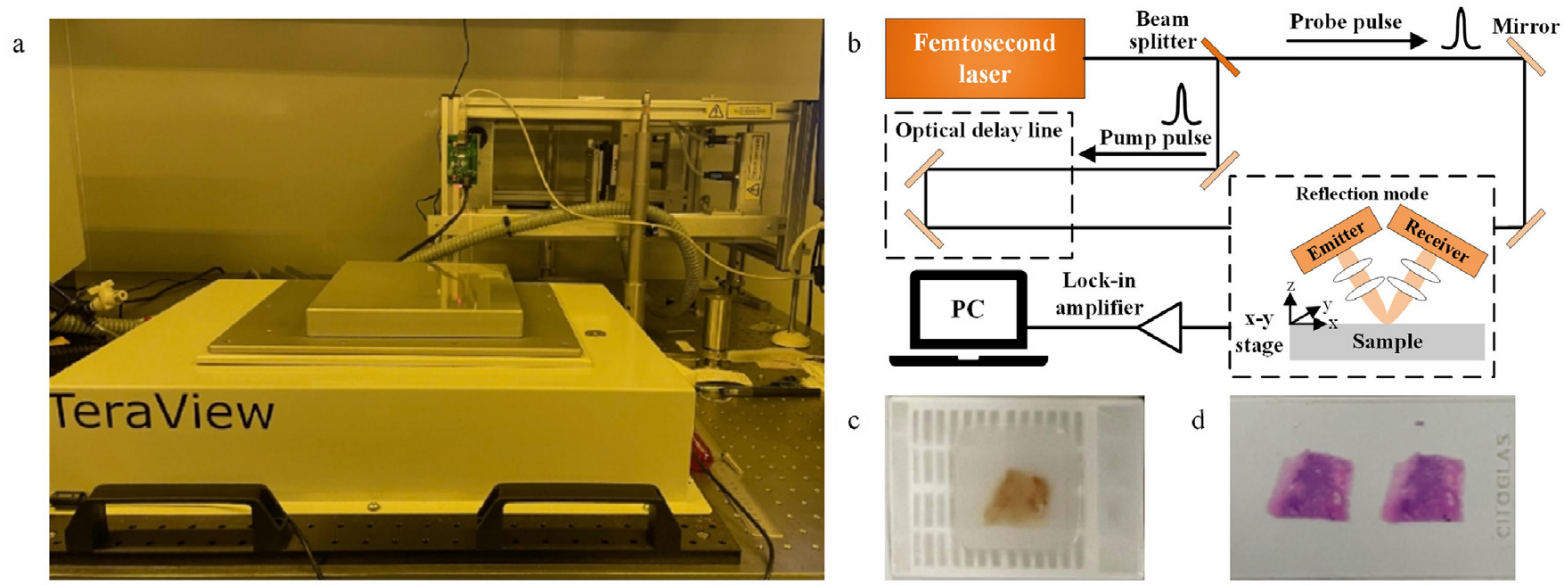
**(a) Reflected THz-TDS system; (b) the schematic diagram of reflected THz-TDS system; (c) sliced cancer tissue; (d) stained slices of cancer tissue**.

As schematically illustrated in Fig. 1, the standard wax block-embedded cancer pathology samples are examined by reflected THz-TDS system in the experiment. THz-TDS system comprises femtosecond laser, emitter and detector, delay control system, and X-Y motion platform. Femtosecond laser emits short bursts of light with durations in the femtosecond range. THz emitter converts the optical pulses from the femtosecond laser into THz radiation. The THz detector receives THz signals reflected back from the sample and convert THz signals into electrical signals. Delay control system controls the timing of the optical and THz pulses to precisely measure THz signals. X-Y motion platform system allows for precise positioning of the sample during the measurement.

As a non-contact imaging technique, THz testing involves several essential calibrations and measurement setup such as THz reference signal, focus position, sample focusing, and spatial step sampling. The THz signal length is configured to *N* = 2048. Notably, all cancer sample measurements are conducted under the same experimental conditions.

### 2.2 THz signals datasets based on cancer tissue

To obtain sufficient THz signals of cancer tissue, four standard wax block-embedded cancer pathology samples, including two breast invasive ductal carcinoma samples, skin high-grade squamous cell carcinoma sample and verrucous carcinoma sample, are scanned by the THz-TDS system to collect THz signals. Then, the collected THz signals are labeled and divided into two distinct datasets, as summarized in Table 1. One dataset is used for the DECANet to identify cancer type, while the other includes positional data for each sampling point, enabling visualization of cancer tissue with pixel-level resolution.

**Table 1:** **The THz signals dataset used for DECANet and imaging**.

Cell type	Label	The dataset used for DECANet			Imaging dataset
		Training dataset	Validation dataset	Testing dataset	
No cell	0	63242	8686	8754	/
Normal cell	1	17251	1745	1696	/
Skin high-grade squamous cell carcinoma	2	3032	345	351	/
Verrucous carcinoma	3	2096	214	222	/
Breast invasive ductal carcinoma	4	14070	1545	1549	/
Total	/	99691	12535	12572	124788

### 2.3 DECANet-based THz detection system for cancer tissue

**Fig. 2:**
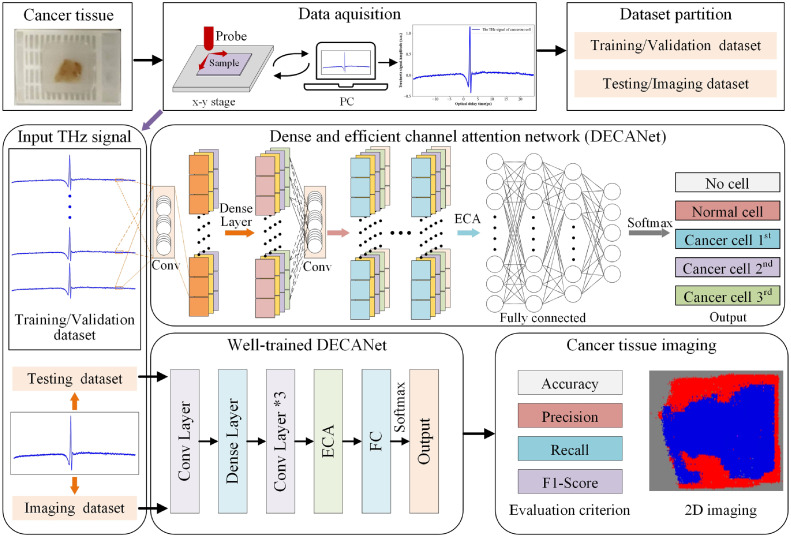
**THz characterization system for cancer tissue**.

As illustrated in Fig. 2, DECANet-based THz detection system for cancer tissue consists of three stages: dataset construction, cancer tissue classification and cancer tissue imaging, as detailed below.

a) Dataset construction. We utilized the THz-TDS system to measure ex-vivo cancer pathology samples. Subsequently, the collected THz signals are labeled and organized into a series of datasets.

b) Model training. Based on the training strategy, the training and validation dataset is used for DECANet to train the model and evaluate the classification performance. Throughout this procedure, temporal THz signals serve as model inputs, while the outputs are the predicted labels of THz signals based on cancer tissue.

c) Cancer tissue imaging. After completing the network training, the well-trained DECANet is fed with the test dataset to obtain the classification results. Subsequently, the DECANet-based THz detection system encodes the predicted labels based on imaging dataset as pixel values, which enables not only accurate identification of cancerous tissues but also provides the location of cancerous regions.

**Fig. 3:**
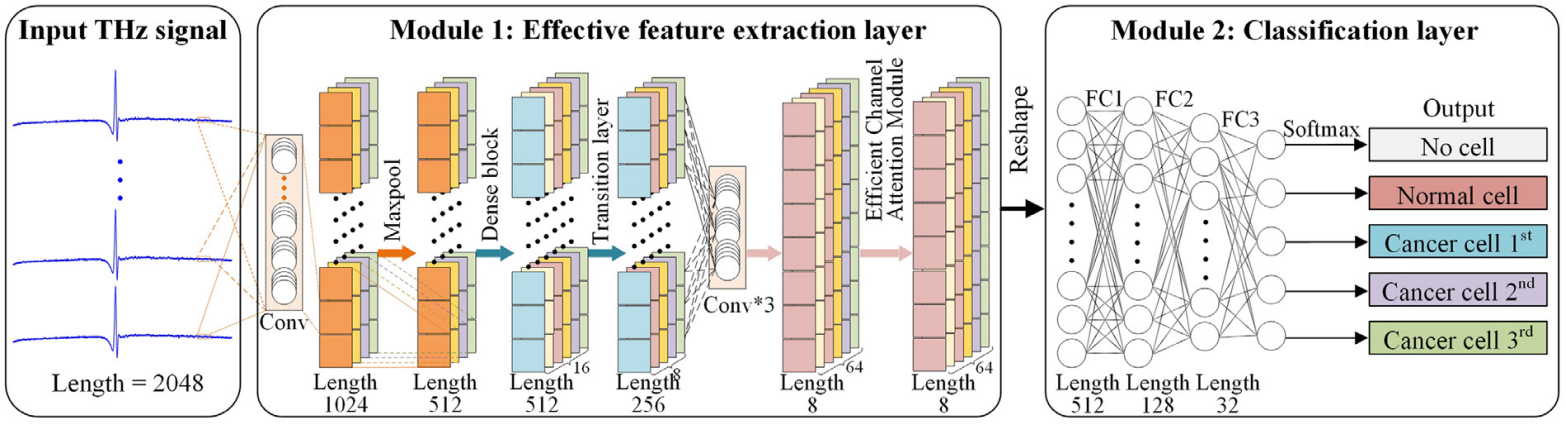
**The overall architecture of proposed DECANet framework**.

Besides, dense and efficient channel attention network (DECANet) framework is proposed to distinguish THz signals from normal tissue and cancer tissue. As illustrated in Fig. 3, DECANet comprises two modules, which include the effective feature extraction layer and classification layer. In the effective feature extraction layer, Conv layer, Dense Block and Transition layer are adopted to reutilize useful features and learn more compact models with few parameters while following a simple joining rule. Then, valuable channel features will be effectively derived through the Conv Layers and efficient channel attention module to improve the classification accuracy. In classification layer, a fully connected layer (FC) combined with a Softmax classifier is utilized to categorize the extracted features. The entire DECANet framework has strong feature extraction capabilities, operating in an end-to-end fashion, and can be easily implemented to assist the accurate evaluation of cancerous tissues by medical professionals.

#### 2.3.1 Effective feature extraction layer

The feature extraction layer first utilizes a regular convolutional layer to capture meaningful features at different scales and abstract levels from one-dimensional THz signal. Then, Dense Block is employed to involve dense connections within the network, which facilitates feature propagation and reutilization to enhance feature representation, and the Transition layer controls the size of feature maps through pooling or convolutional operations, which can reduce computational complexity and prevent the model overfitting. Three convolutional layers are responsible for capturing features at various scales and abstract levels in the output feature map of the Transition layer. This allows the model to extract diverse information to generate a comprehensive and informative feature representation. After the initial feature extraction through three convolutional layers, the effective channel attention mechanism is employed to emphasize important channel features while suppressing less useful ones.

#### 2.3.2 Conv layer

**Fig. 4:**
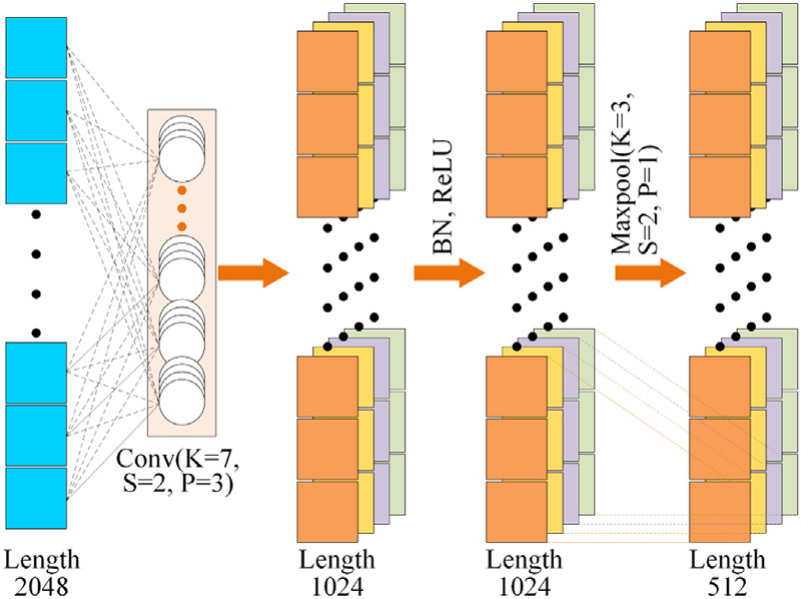
**The detailed architecture of Conv layer**.

The regular convolutional layer is a commonly used feature extraction operation in deep learning, which extracts local features from the original one-dimensional THz signal by convolutional operations. In the effective feature fusion layer, the Conv layer plays a crucial role and includes the following operations: convolution, BN, ReLU, and Maxpooling, as shown in Fig. 4.

The convolution operation performs element-wise multiplication and summation between the convolutional kernel and the input one-dimensional THz signal. Then the slide of convolutional kernel over different positions will generate the output feature maps. BN is introduced to enhance the training stability and accelerate the convergence. ReLU is a common activation function that introduces the non-linearity. It sets negative input values to zero while keeping non-negative values unchanged, thereby increasing the expressive power of the network and mitigating the problem of vanishing gradients to some extent. Maxpooling reduces the dimensionality of feature maps, captures dominant features, and enhances the translation invariance of the network. By combining these operations in the Convolution layer, effective feature extraction and representation can be achieved to enable the network to leverage essential patterns in the THz signal.

#### 2.3.3 Dense layer

**Fig. 5:**
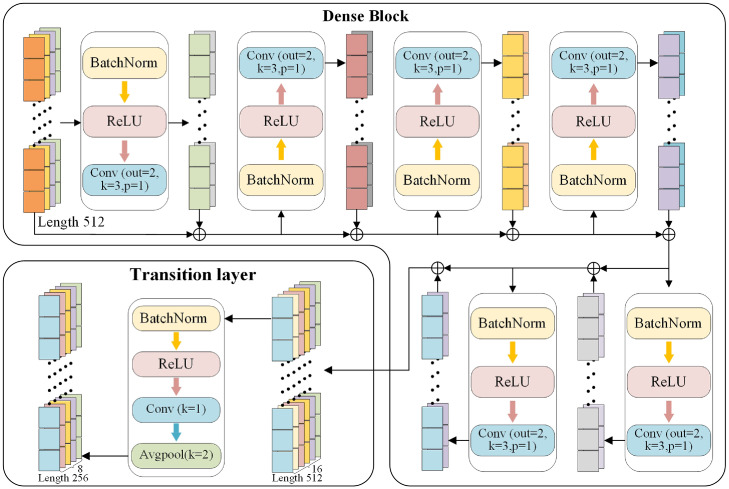
**The detailed architecture of dense layer**.

A complex and powerful deep learning model can be constructed by repeatedly stacking and combining Conv layers. These layers act as feature extractors at different levels and form deep learning network, in which the rich features are amplified by adding more stacked layers (or increasing depth). However, blindly stacking more layers in the network without taking the issue of vanishing/exploding gradients into account does not guarantee the output performance. On the contrary, it introduces unnecessary complexity and redundancy and results in significant increases in the training and inference time. To address this challenge, a deep residual learning framework was proposed to maintain the accuracy gains from greatly increased depth in ResNet [24], which substantially improved the performance compared to previous networks. However, the improvement of deep residual networks relies on stochastic depth that involves randomly dropping layers during the training. It should be noted that not all layers require the stochastic depth to generate a significant amount of redundancy. Further, Huang et al. [25] have proposed dense convolutional layer, which is designed to alleviate the issue of vanishing gradients and substantially reduce the model parameter number. Due to the complexity of THz signals, this strategy offers promising potential for efficiently extracting cancer-related information from complex THz signals. Therefore, we incorporate a dense connectivity pattern into the standard convolution, introducing a dense convolutional layer to strengthen the feature propagation and reutilization (Fig. 5).

In Fig. 5, dense layer incorporates Dense Block and Transition layer. The former establishes connections between each layer in a feed-forward fashion to improve the information transfer between layers and preserving low-dimensional features. It is important to highlight that all the input layers have feature maps with the same size. The $l^{th}$ layer receives the feature-maps of all preceding layers $\left(x_{0},\cdots ,x_{l-1}\right)$ as input.

(1) $$x_{l}=H_{l}^{\prime }\left(\left[x_{0},x_{1},\cdots ,x_{l-1}\right]\right)$$

where $\left[x_{0},\cdots ,x_{l-1}\right]$ refers to the concatenation of the feature-maps produced in layers 0,1,⋯,l−1, $H_{l}^{\prime }\left(\cdot \right)$ defines a function composed of BN, followed by a ReLU and a Conv with kernel_size = 3, which is commonly referred to pre-activation as it applies ReLU before the BN.

In traditional networks, it is common for each layer within a block to replicate all preceding feature maps as they progress from layer to layer. However, in a dense block, it can access the overall state from any point within the block, provided that all earlier feature maps inside the block have been recorded. The growth rate is a critical factor in determining the extent of new information each layer adds to the overall network state. Specifically, each layer adds *k* feature-maps of its own to this state. In this dense block, we set the network growth rate as *k* = 2 so that the dense block has narrow feature layers and few parameters. If each function $H_{l}$ produces *k* feature maps, the $l^{th}$ layer input feature map can be determined as $k_{0}+k\left(l-1\right)$, where $k_{0}$ is the channel number of input layer. After outputting the feature map from the dense block, it is passed through the transition layer, which consists of several operations including BN, ReLU, convolution and average pooling (Avgpool). The convolutional layer applies convolutional filters to the input to generate a small set of feature maps with reduced channel dimensions. This operation helps to compress and condense the information in the feature map. Additionally, by performing Avgpool with two steps, the feature map length is reduced by half to further decrease the model complexity. This down sampling process helps to capture the most relevant and representative features while reducing the computational memory requirements of the model.

In summary, the Dense Block promotes extensive information flow between layers and enables the feature reutilization to generate compact models with few parameters. The Transition Layer plays a crucial role in reducing the channel number and model complexity, which further refine and down sample the feature maps. The introduction of Dense Block and Transition Layer increases the potential to accurately extract cancer-specific information from complex THz signals. By effectively preserving low-dimensional features and enhancing the information flow, this approach enables the proper classification of cancer cells by capturing the cancer-related features.

#### 2.3.4 Efficient channel attention (ECA)

Effective channel attention [26] is a technique that aims to enhance the representation power of the model by selectively focusing on informative channels in the feature map. Unlike complex attention modules that increase the model complexity for compensating the performance, the ECA module introduces a minimal number of additional parameters and requires negligible computations. Despite its lightweight nature, the ECA module delivers significant performance benefits. To leverage the advantages of ECA module, it is incorporated into feed-forward convolutional neural networks. Specifically, it is placed behind the convolutional layer to learn effective channel attention and suppress less useful channel features while maintaining a low model complexity.

**Fig. 6:**
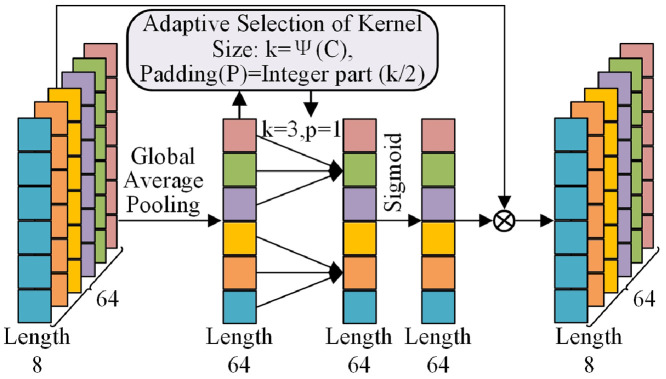
**The ECA architecture**.

ECA module can be efficiently implemented by convolution without dimensionality reduction, as shown in Fig. 6. Initially, supposing that given feature maps $X=\left[x_{1},x_{2},\cdots ,x_{C}\right]\in R^{C\times L}$, Global Average Pooling layer is employed to capture the channel-wise global statistic information α. *c*-th element of α can be obtained as

(2) $$\alpha_{c}\left(x_{c}\right)=\frac{1}{L}\sum_{i=1}^{L}x_{c}\left(L\right)$$

where $x_{c}\left(L\right)$ represents the pixel value of the *c*-th feature map $x_{c}$ at position *L*.

Then, one-dimensional convolution is used for local cross-channel interaction. Given the aggregated feature map $F\in R^{C}$ with *C* channels without dimensionality reduction to learn channel attention:

(3) α=σ(W×F)

where *W* denotes parameter matrices with the dimension of *C* and σ(·) is a sigmoid gating function. In order to effectively obtain the discriminant representation across feature channels, it is necessary to model the local cross-channel interaction. So the weight of $F_{i}$ can be calculated by

(4) $$\alpha_{i}=\sigma \left(\sum_{j=1}^{k}w^{j}F_{i}^{j}\right),F_{i}^{j}\in \Omega_{i}^{k}$$

where $\Omega_{i}^{k}$ represents the group of *k* neighboring channels of $F_{i}$. Besides, this local aggregation is precisely achieved using a convolution layer (kernel size = *k*)

(5) $$\alpha =\sigma (conv_{1D}\left(F\right))$$

Depending on the channel dimension *C*, kernel size *k* and the padding *p* can be adaptively determined by

(6) $$k=\psi \left(C\right)={\left|\frac{\log_{2}\left(C\right)}{\gamma }+\frac{b}{\gamma }\right|}_{odd}$$

(7) $$\begin{array}{c} p=\text{Integer}\;\text{part}\left(\frac{k}{2}\right) \end{array}$$

where ${\left|t\right|}_{odd}$ indicates the odd number nearest to *t*. In our experiments, γ and *b* are set to 2 and 1, respectively. According to Eq. 6 and 7, *k* and *p* are equal to 3 and 1 for the given 64 channels.

#### 2.3.5 Multi-classification layer

After the effective feature extraction layer, the obtained multiscale features are reshaped into a one-dimensional feature representation. To further process these features and obtain the final classification result, a FC layer is employed to transform one-dimensional features into a final feature map, which captures the relevant information for the classification task. To assign a probability distribution to each category, a Softmax classifier is applied to compute the conditional probability for each category based on the transformed features. By utilizing the FC layer and Softmax classifier, the model can assign a probability value to each class, indicating the likelihood of a given THz signal belonging to a specific cell type. This enables accurate classification of different cell types based on the THz signal analysis.

#### 2.3.6 Class-balanced cross-entropy loss function

The Loss Function is employed to guide the learning process towards minimizing the difference between predicted values and labels in DL. For example, Cross-entropy loss function has been commonly used to optimize the parameters of neural network. However, the cross-entropy function is sensitive to data distribution and category imbalance. Due to the inherent heterogeneity of cancer samples, different types of cancer cells exhibit various prevalence in real biological tissues. For instance, certain cancer types may be more prevalent due to their aggressive nature or high occurrence rates, while other types may be relatively rare. As a consequence, when constructing a THz signal dataset from cancer slices, the number of instances for each specific cancer cell exhibit a highly uneven distribution. This imbalance poses challenges for the model training and evaluation, which needs appropriate techniques to mitigate its impact on the model performance.

Therefore, the class-balanced cross-entropy loss function is considered to address the problem of category imbalance in cancer THz datasets. The proposed loss function in DECANet considers the imbalanced distribution of different classes and assigns appropriate weights to each category during the training, which can be written mathematically as

(8) $$L_{balancedloss}=-\frac{1}{N}\sum_{i=1}^{N}\sum_{j=1}^{M}\omega_{j}y_{i,j}\log\left({\hat{y}}_{i,j}\right)$$

where *N* and *M* are the number of training examples and classes, respectively; ${\hat{y}}_{i,j}$ denotes the predicted probability; $\omega_{j}$ is the weight for class *j*, and can be calculated as

(9) $$\omega_{j}=\frac{1}{n_{i}}$$

where $n_{i}$ represents the sample count for class *i*.

By assigning large weights to underrepresented classes and small weights to overrepresented classes, it helps to alleviate the bias towards major classes and focus more on learning from minor classes. This approach enables the model to improve the overall accuracy and effectiveness in handling imbalanced cancer THz datasets.

## 3 Results and discussion

The DECANet is implemented on the PyCharm Community Edition 2021.3.2 × 64 and NVIDIA GeForce RTX 3090. The initial learning rate is employed as $1\times 10^{-3}$ and the learning rate decreases by 0.2 every 20 epochs with batch size = 16 and epoch = 100. To minimize the classification randomness, all experiments are conducted three times to calculate the average classification accuracy.

### 3.1 Performance evaluation of DECANet

**Fig. 7:**
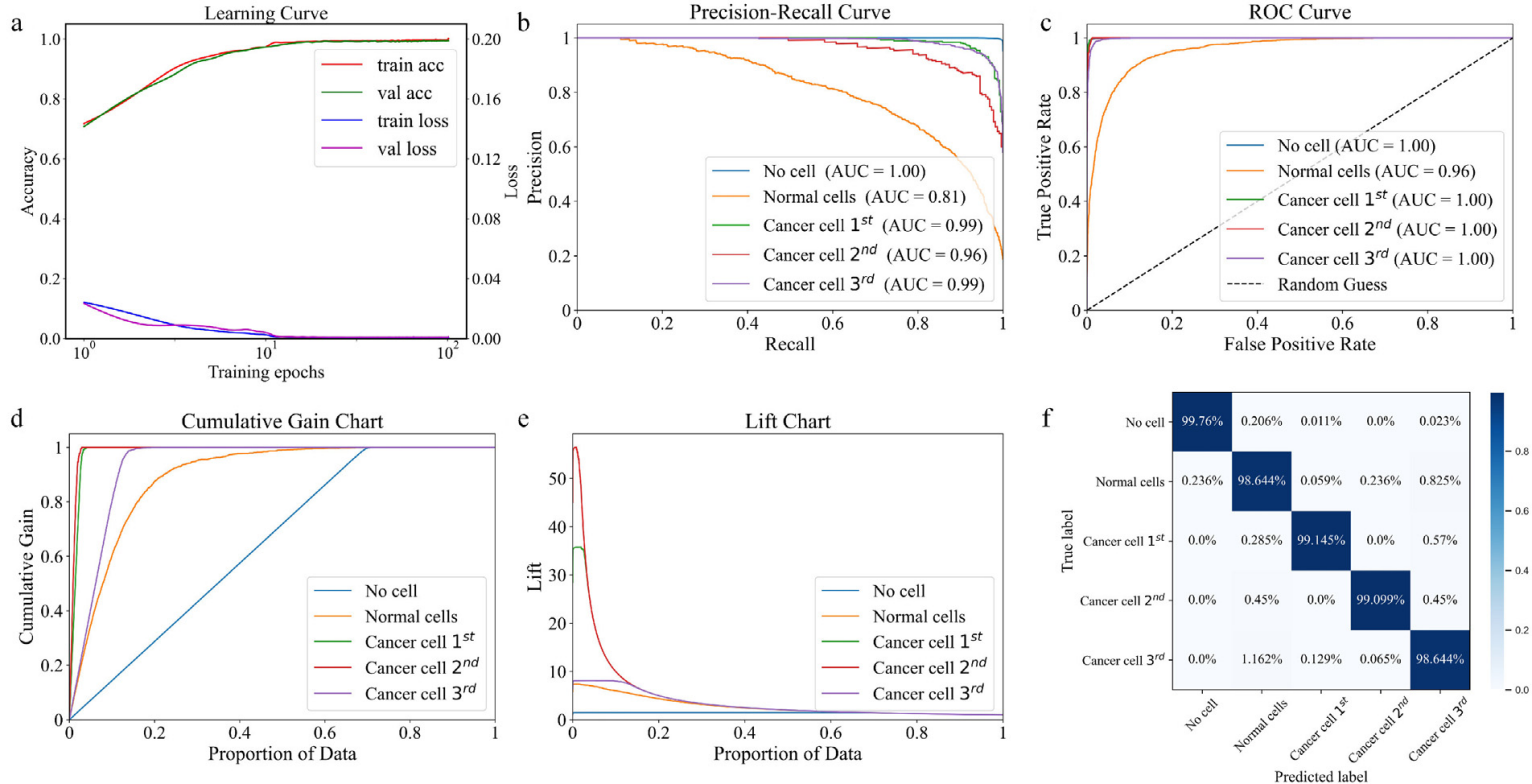
**(a) Learning Curve, (b) PR Curve, (c) ROC Curve, (d) Cumulative Gain Chart and (e) Lift Chart of DECANet model; (f) The confusion matrix for the testing dataset**. The cancer $1^{\mathrm{st}}$is breast invasive ductal carcinoma, the cancer $2^{\mathrm{nd}}$ is skin high-grade squamous cell carcinoma sample and the cancer $3^{\mathrm{rd}}$ is verrucous carcinoma.

The Fig. 7 presents various evaluation metrics for the DECANet model, including the learning curve, Precision-Recall Curve (PR Curve), Receiver Operating Characteristic Curve (ROC Curve), cumulative gain chart, and lift chart, along with confusion matrix for the testing dataset. Fig. 7a illustrates the learning curve of DECANet model and the results clearly demonstrate the effectiveness of proposed network on accurately classifying various cell types, including no cell, normal cell, and different types of cancer cells. The plot compares the accuracy and loss trends for both the training and validation datasets. As the training progresses, the loss steadily decreases for both datasets, with the training loss approaching a stable value near zero and the validation loss decreasing at a slower pace, indicating effective learning by the model. Fig. 7b illustrates the PR curve, which evaluates a deep learning model's classification accuracy and its efficacy in identifying relevant instances, by assessing the proportion of true positives (TP) among predicted positives and the ratio of actual positives correctly identified. The movement of PR curve toward the top-right corner indicates the model's capability to detect positive samples efficiently. The area under curve (AUC) quantifies this performance, with higher values indicating better overall precision and recall. Besides, the DECANet model maintains high precision even with a low recall, indicating that it minimizes false positives, which is particularly important when the number of positive samples is scarce. Fig. 7c shows the ROC curve, where offers a visual depiction of a model's balance between detecting TP and avoiding false positives (FP) across various thresholds. The AUC serves as a measure of the model's discriminative power, with the curve approaching the top-left corner, signifying that DECANet achieves excellent classification performance. A higher AUC indicates that the model is effective at differentiating between various types of signals. Fig. 7d presents the Cumulative Gain Chart, which demonstrates the efficacy of a model by sorting its prediction results. The horizontal axis represents the cumulative percentage of data points sorted from highest to lowest based on the model's predicted probability, while the vertical axis shows the cumulative percentage of actual positive numbers within data points. It can be observed directly from the chart that the curve rapidly ascends to the upper-left corner, indicating that selecting just a small fraction of the highest-scoring data points identified by the model can capture a significant number of TP, demonstrating the robust discriminative capacity of DECANet model. The Lift Chart illustrates the enhancement in the proportion of positive samples relative to random selection, expressed as the ratio of cumulative gain at a given data percentage to the expected gain by chance. In Fig. 7e, a higher lift value indicates that the model's efficiency in selecting potential positive samples significantly surpasses random selection, demonstrating the ability to prioritize high-value (positive) samples more effectively. Subsequently, the confusion matrix in Fig. 7f provides a comprehensive summary of classification results for a detailed analysis. By examining the diagonal elements of the matrix that represent correctly classified samples and the off-diagonal elements that indicate the misclassifications, we can gain insights into the accuracy of DECANet framework and identify specific areas that need further improvements. In Fig. 7f, the DECANet model accurately classifies all samples in the testing dataset into the categories of no cell, normal cell, and different types of cancer cells. It demonstrates the model's capability to effectively recognize and distinguish various cancerous cell types.

Besides, we have supplemented classification report based on the test dataset in Table 2. The high precision indicates that a significant proportion of the samples predicted as positive by the model are correctly labeled, meaning there are few FP. The high recall score signifies that DECANet successfully identifies most of the actual positive samples, demonstrating excellent performance in capturing relevant instances, with fewer false negatives. And the high F1 score suggests that DECANet maintains a good balance between accuracy and coverage, which is particularly crucial when dealing with imbalanced datasets. The classification report in Table 2 shows metrics across all categories, indicating that the model possesses strong generalization capabilities across various types of THz signals.

**Table 2:** **The classification report on the test dataset**.

Cell type	Label	Precision	Recall	F1-score	Support
No cell	0	99.95%	99.76%	99.86%	8754
Normal cell	1	97.78%	98.64%	98.21%	1696
Verrucous carcinoma	2	98.86%	99.15%	99.00%	351
Skin high-grade squamous cell carcinoma	3	97.78%	99.10%	98.43%	222
Breast invasive ductal carcinoma	4	98.77%	98.64%	98.71%	1549

### 3.2 Ablation experiments of DECANet

The ablation experiments are conducted to examine the impact of different strategies on the performance of DECANet model. The ablation experiments systematically assessed the effect of removing specific modules from the DECANet and analyzed their contributions to the overall model performance. To facilitate a comprehensive evaluation, key performance metrics including Accuracy, Cohen's Kappa Coefficient, and so on. As illustrated in Table 3, the base CNN model achieved an accuracy of 0.9499, demonstrating solid classification performance. Introducing the Dense Layer increased accuracy to 0.9728, while adding the ECA module further improved it to 0.9775. Ultimately, the complete DECANet, incorporating all modifications, reached an impressive accuracy of 0.9944, highlighting the robustness of DECANet. The Cohen's Kappa Coefficient serves as a valuable indicator of classification accuracy that considers the consistency between actual labels and predicted results, and adjusts for random agreements, with higher values indicating better consistency. Notably, the Cohen's Kappa increased from 0.8970 for the CNN to 0.9884 for the full DECANet, highlighting a substantial improvement in agreement between predicted values and labels, highlighting the reliability of DECANet. The Matthews Correlation Coefficient (MCC) offers a comprehensive assessment of classifier performance, balances the distribution of different classes, and considers all types of errors, thereby providing a fair measure of overall model performance. In our results, the MCC increased from 0.8980 for the CNN to 0.9884 for the DECANet, reflecting the model's enhanced ability to handle all types of errors effectively. Additionally, the Jaccard Index quantifies the overlap between predicted positives and TP. A Jaccard Index of 0.8022 for the base CNN model improved to 0.9772 with the complete DECANet, indicating a strong performance in accurately identifying relevant instances within the dataset.

**Table 3:** **Ablation experiments of DECANet on the test dataset**.

Model	Accuracy	Cohen's Kappa	MCC	Jaccard Index
CNN	0.9499	0.8970	0.8980	0.8022
+Dense Layer	0.9728	0.9435	0.9435	0.9028
+ECA	0.9775	0.9531	0.9531	0.9154
+Loss	0.9944	0.9884	0.9884	0.9772

**Fig. 8:**
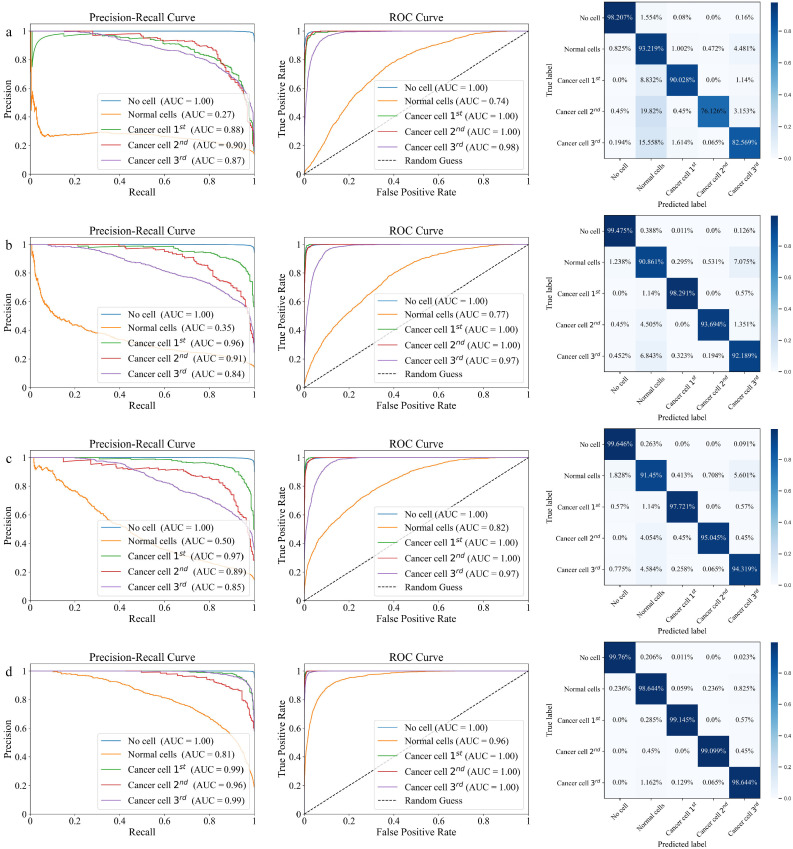
**The PR Curve, ROC Curve, and confusion matrix for ablation experiments on the testing dataset**. (a) CNN; (b) +Dense Layer; (c) +ECA; (d) +Class-balanced Cross-entropy Loss.

Besides, Fig. 8 showcases the performance of different configurations of the DECANet model through PR Curve, ROC Curve, and confusion matrix, with (a) the CNN model, (b) the addition of a Dense Layer, (c) the incorporation of ECA, and (d) the use of Class-balanced Cross-entropy Loss. The PR Curve of Fig. 8a-d,highlights the trade-off between precision and recall for each model configuration, and we observe that adding the Dense Layer, ECA, and Class-balanced Cross-entropy Loss results in a gradually increase in the AUC of PR Curve, thereby indicating improved identification of relevant signals. Specifically, the inclusion of these enhancements allows the model to better detect positive samples. The ROC curve further highlights the model's ability to distinguish TP from FP across different thresholds. As the ROC curve moves from Fig. 8a-d, it gradually approaches the top-left corner, indicating that the model effectively minimizes FP while maximizing TP detection, which is particularly important in tasks like cancer classification where FP may lead to significant misdiagnosis. A higher AUC suggests that the model has a better discriminatory ability between the positive and negative classes. The ROC curve of DECANet in Fig. 8d consistently stays above the random guessing line, showcasing its strong classification performance across different class scenarios. Additionally, a comparison of the confusion matrices in Fig. 8a-d across different configurations clearly shows that incorporating the Dense Layer, ECA, and Class-balanced Cross-entropy Loss significantly improves the model's classification performance. These enhancements specifically lead to higher accuracy in distinguishing between normal cells and cancer cell samples, while also reducing misclassifications across categories. This highlights the effectiveness of the proposed modifications in boosting the overall performance of the DECANet model. Overall, these results from the ablation experiments convincingly demonstrate that the additions of the Dense Layer, ECA, and Class-balanced Cross-entropy Loss to improved performance metrics, validating the effectiveness of the proposed modifications to the DECANet architecture.

### 3.3 Comparison of recognition performance with ML algorithm

**Fig. 9:**
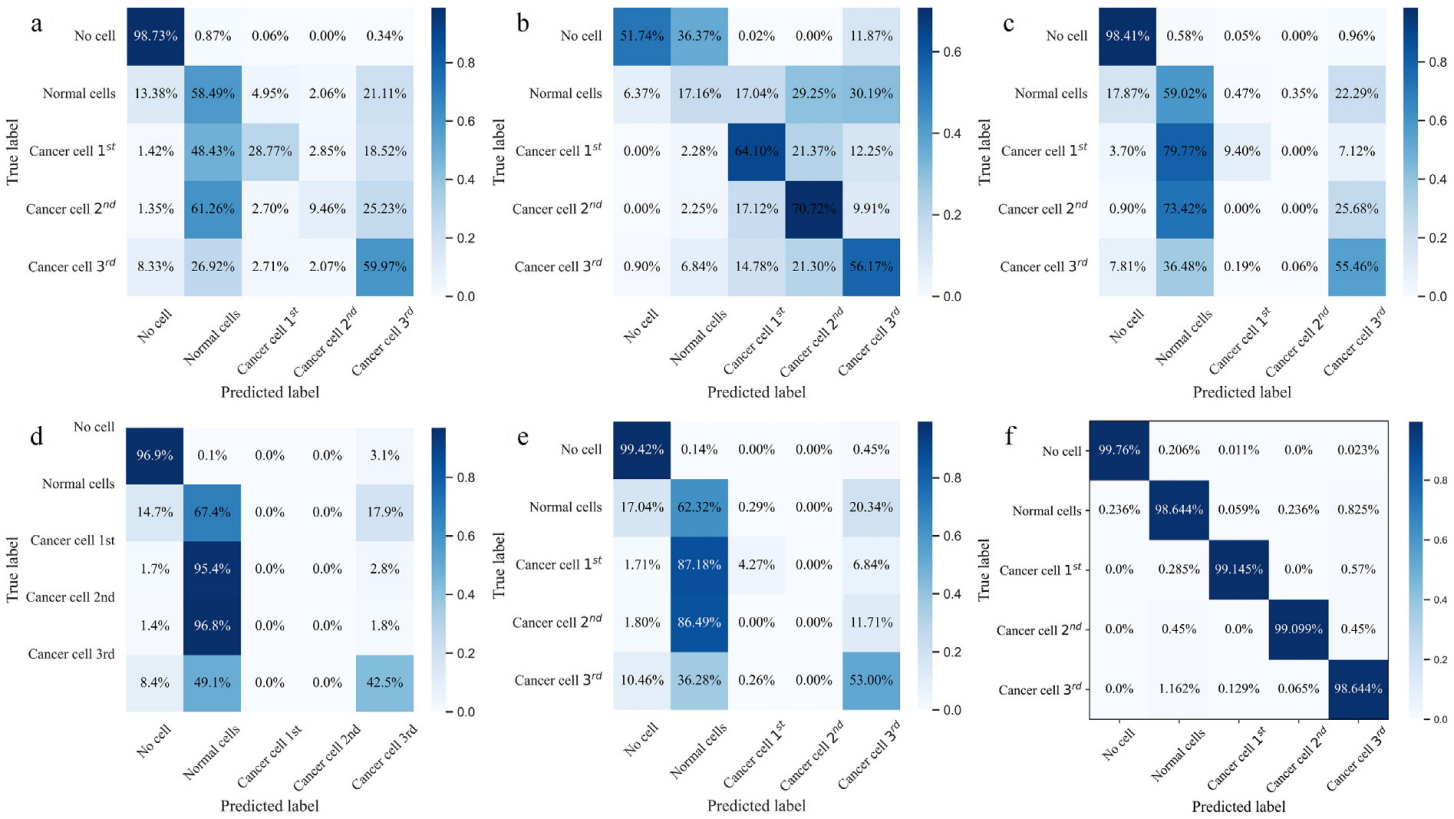
**The confusion matrix for (a) KNN; (b) GNB; (c) LR; (d) DT; (e) SVM and (f) DECANet on the same testing dataset**.

In contrast to DECANet, traditional ML algorithms necessitate the manual selection of relevant features from the original signal for classification tasks. For this study, we employed five classical ML techniques, including K-Nearest Neighbors (KNN), Gaussian Naive Bayes (GNB), Logistic Regression (LR), Decision Tree (DT) and Support Vector Machine (SVM) all based on manually selected features. These manually chosen features include various statistical parameters, as outlined in Table 4. After extracting these statistical parameters, they were fed into the respective ML algorithms, as displayed in Table 5. The results clearly demonstrate that DECANet outperformed traditional methods, achieving highest training and testing accuracy, demonstrating the superiority over manually engineered features for classification. Furthermore, Fig. 9 demonstrates that the confusion matrices for DECANet exhibit significantly higher diagonal values compared to traditional machine learning methods, highlighting its remarkable ability to accurately classify instances across all categories.

**Table 4:** **Equation of statistical parameters for comparison**.

Statistical parameters	Symbol	Equation
Mean	$\bar{x}$	$N^{-1}\sum x$
Standard deviation	$\sigma_{x}$	$\sqrt{{\left(N-1\right)}^{-1}\sum {\left(x-\bar{x}\right)}^{2}}$
Root mean square	$x_{rms}$	$\sqrt{N^{-1}\sum x^{2}}$
Peak-to-peak	Vpp	max(*x*)-min(*x*)
Peak metric	*C*	$\max\left(x\right)/x_{rms}$
Waveform metric	*W*	*x_rms_*/$\bar{x}$
Pulse metric	*I*	max(*x*)/$\bar{x}$
Margin metric	*L*	$\max\left(x\right)/\sqrt{N^{-1}\sum {\left|x\right|}^{2}}$
Steepness metric	*K*	$\sqrt{N^{-1}\sum {\left(x-\bar{x}\right)}^{4}}/\sigma_{x}^{4}$

**Table 5:** **Comparisons with different machine learning models**.

Models	Average accuracy (%)	
	Training dataset	Testing dataset
Statistical parameters + KNN	85.78	85.00
Statistical parameters + GNB	46.80	48.30
Statistical parameters + LR	79.12	83.58
Statistical parameters + DT	80.46	80.02
Statistical parameters + SVM	80.07	84.28
Proposed DECANet	**99.53**	**99.44**

To further validate the superiority of DECANet, other deep learning models including CNN, BiLSTM, ResNet and Transformer are also compared on the same dataset, as illustrated in Table 6. It is evident that DECANet consistently outperforms the other models across key performance metrics, achieving an impressive accuracy of 0.9944. Furthermore, DECANet exhibits a remarkable Cohen's Kappa of 0.9884 and a MCC of 0.9884, reflecting a high degree of consistency and reliability in its predictive capabilities. In contrast, CNN, BiLSTM, ResNet and Transformer present comparatively lower values, with Cohen's Kappa scores of 0.8970, 0.8800, 0.8938 and 0.9027, and MCC scores of 0.8980, 0.8804, 0.8955 and 0.9037. Collectively, these results underscore the superior classification performance of DECANet.

**Table 6:** **Comparisons with different DL models**.

Models	Accuracy	Cohen's Kappa	MCC	Jaccard Index
CNN	0.9499	0.8970	0.8980	0.8022
BiLSTM	0.9424	0.8800	0.8804	0.7401
ResNet	0.9500	0.8938	0.8955	0.8212
Transformer	0.9786	0.9027	0.9037	0.8197
DECANet	0.9944	0.9884	0.9884	0.9772

**Fig. 10:**
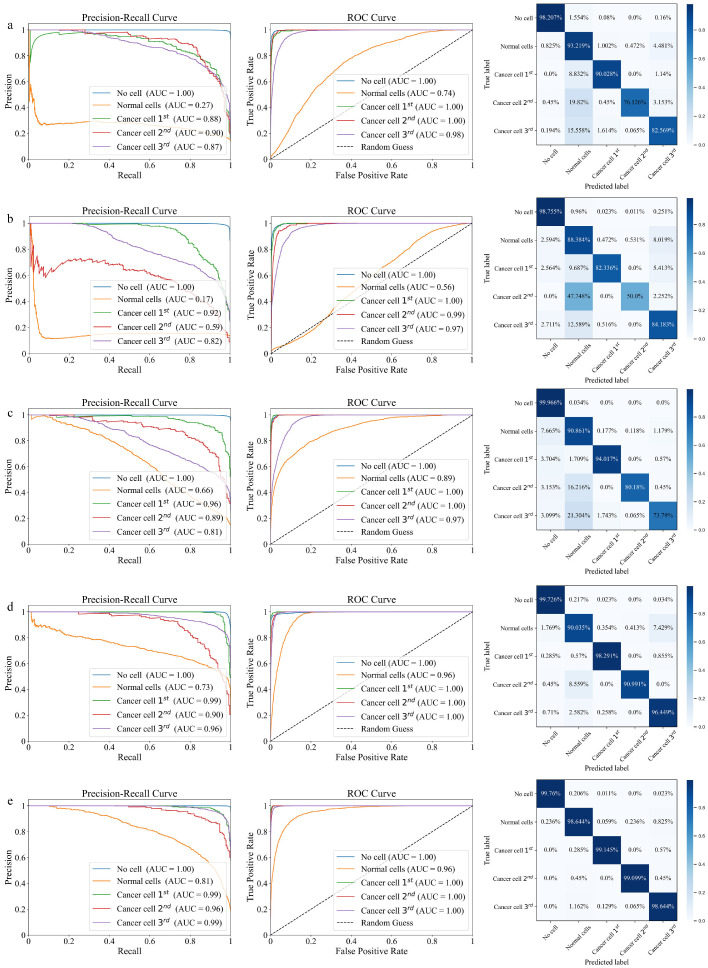
**The Precision-Recall Curve, ROC Curve, and confusion matrix for (a) CNN; (b) BiLSTM; (c) ResNet; (d) Transformer and (e) DECANet on the testing dataset**.

The comprehensive performance evaluation of various models is comprehensively illustrated in Fig. 10, which showcases the Precision-Recall Curves, ROC Curves, and corresponding confusion matrices. Fig. 10a-e clearly demonstrate a significant improvement in classification performance when moving from traditional models, such as CNN, BiLSTM, ResNet, and Transformer, to the proposed DECANet architecture. The PR Curves in Fig. 10a-d clearly underscore this improvement, with DECANet (Fig. 10e) demonstrating a significantly better balance between precision and recall across various thresholds compared to traditional models. Specifically, DECANet shows superior capability in detecting relevant signals, as demonstrated by its shift toward the top-right corner of the PR Curve, indicating a better balance between precision and recall. Furthermore, the ROC Curves in Fig. 10a-d reinforce the PR curve findings, with DECANet (Fig. 10e) consistently demonstrating a higher TP rate and a lower FP rate, significantly outperforming the other models that hover closer to the random guessing line. This contrast emphasizes DECANet's robust classification performance, particularly in multiclass scenarios, where traditional models fall short. The confusion matrices of DECANet (Fig. 10e) reveal significantly higher diagonal values compared to those of CNN, BiLSTM, ResNet and Transformer in Fig. 10a-d, highlighting its superior ability to accurately classify instances across all categories. The high diagonal values represent correctly classified instances, while the lower misclassification rates emphasize the model's reliability. In contrast, the traditional models show more frequent misclassifications, highlighting their limitations. Taken together, these results not only validate the proposed modifications but also clearly demonstrate that DECANet outperforms the other models in achieving superior classification accuracy on the testing dataset.

### 3.4 Imaging of cancer samples

**Fig. 11:**
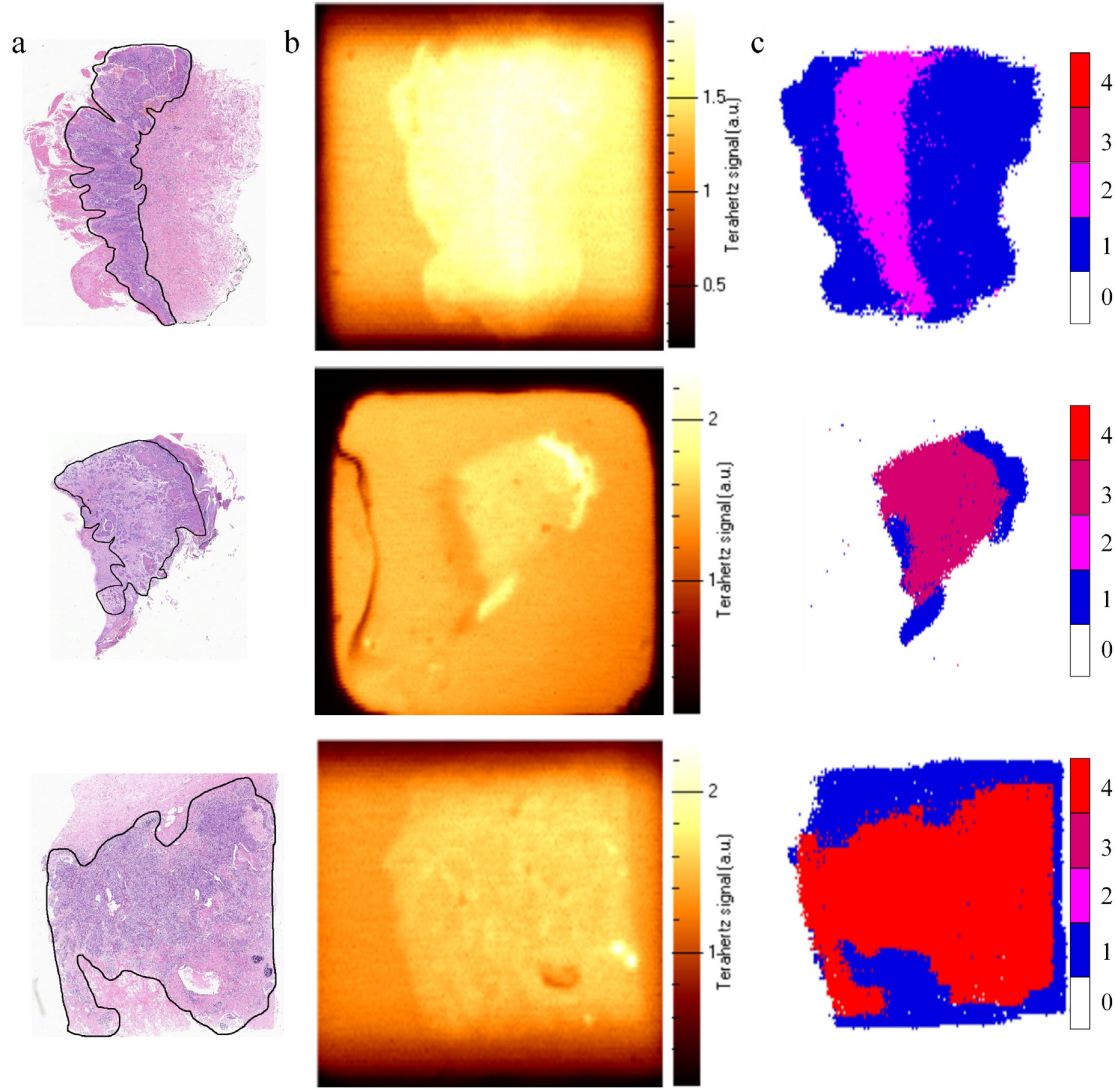
**2D imaging for the imaging dataset.** (a) Microscopic images, (b) LPTP images, (c) DECANet-based 2D images for biopsy samples of Verrucous carcinoma, Skin high-grade squamous cell carcinoma and Breast invasive ductal carcinoma.

Fig. 11a shows the microscopic images for cancer tissue biopsy samples and Fig. 11b shows the C-scan images of cancer samples using the feature-based local peak-to-peak (LPTP) imaging algorithm, which is widely utilized in THz time-domain imaging. Different colors represent different cell types. According to the LPTP algorithm shown in Fig. 11b only the outline of the tissue sample is visible which makes it difficult to distinguish between cancerous and normal tissues. Besides, the pixel value of each sampled point is determined by encoding the pixels according to the cell type. By doing so, the imaging dataset is input into the well-trained DECANet to achieve the characterization of cancer tissue samples, as illustrated in Fig. 11c. In contrast, Fig. 11c achieves pixel-level imaging resolution without the need for intricate manual feature extraction while distinctly delineating cancerous tissue boundaries. This allows simultaneous display of both the position and shape information of cancer tissue and demonstrate the wide applicability of THz imaging for the biomedical applications.

## 4 Conclusion

In this paper, we present a DECANet-based characterization system, transforming the localization and imaging of cancer tissue samples into a multi-class deep learning task. Our approach achieves high-precision classification of THz signals across different malignancies and enables the automated identification of cancerous samples. During the classification process, we design a dense convolution layer to strengthen feature propagation and take feature reutilization into account, thereby improving the feature extraction capacity. In addition, the lightweight ECA module is placed behind the convolutional layer in feed-forward convolutional neural networks to learn effective channel attention and suppress less useful channel features with low model complexity. We also propose the class-balanced cross-entropy loss function to solve the problem of category imbalance in cancer THz datasets. The ablation experiments on the DECANet concludes that each module can improve the accuracy on the detection of cancer cell in the cancer tissue. Compared to tradition ML Algorithms, the accuracy of training and testing dataset by proposed DECANet model are 99.83% and 99.74%, respectively. The accuracy of testing dataset by proposed DECANet model is 99.74%, which is larger than that of other deep learning models. During the imaging of cancer tissue samples, the classification coding strategy is employed to obtain cancer tissues images. Unlike traditional THz nondestructive LPTP imaging algorithm, this imaging method provides position and shape information with high-precision resolution for various cancer tissue. Hence, the DECANet-based characterization system delivers an end-to-end, universal THz characterization approach that can guide clinical treatment decisions in cancer evaluations.

## Declaration of competing interests

The authors declare that they have no conflicts of interest in this work.
